# Retraction Note: HOXD9 promotes epithelial–mesenchymal transition and cancer metastasis by ZEB1 regulation in hepatocellular carcinoma

**DOI:** 10.1186/s13046-024-03171-z

**Published:** 2024-08-30

**Authors:** Xiupeng Lv, Linlin Li, Li Lv, Xiaotong Qu, Shi Jin, Kejun Li, Xiaoqin Deng, Lei Cheng, Hui He, Lei Dong

**Affiliations:** 1 Department of Radiation Oncology, First Affiliated Hospital, Dalian Medical University, Dalian, 116001 China; 2 Department of the 4th Internal Medical, Liaoning Cancer Hospital & Institute, Shenyang, 110042 China; 3 Department of Pathology, Second Affiliated Hospital of Dalian Medical University, Dalian, 116027 China; 4 Department of Second Neurology, The First Affiliated Hospital of Dalian Medical University, Dalian, 116001 China; 5 Department of Laparoscopic Surgery, First Affiliated Hospital of Dalian Medical University, Dalian, 116001 China; 6 Department of Radiation Oncology, First Affiliated Hospital of Dalian Medical University, Dalian, 116001 China

**Retraction Note: J Exp Clin Cancer Res 35**,** 133 (2015)**

10.1186/s13046-015-0245-3

The Editor-in-Chief has retracted this article at the corresponding author’s request. After publication, concerns were raised that the data presented in this article appear highly similar to a number of other publications [1–5].

The corresponding author has stated that the experiments were performed by a third party, where the data may have been mismanaged.

Lei Dong agrees to this retraction. None of the other authors have responded to any correspondence from the publisher about this retraction.
